# Effects of Stress on Mothers of Hospitalized Children in a Hospital in Iran

**Published:** 2012

**Authors:** Tayebeh HASAN TEHRANI, Mohammad HAGHIGHI, Hasan BAZMAMOUN

**Affiliations:** 1 Member of Nursing, Research Center for Child and Maternity Cares, Hamedan University of Medical Sciences, Hamedan, Iran; 2 Assistant Professor of Psychiatry, Department of Psychiatry, Farshchian Hospital, Hamedan University of Medical Sciences, Hamedan, Iran; 3 Assosciate Professor of Pediatric Gastroenterology, Department of Pediatrics, Besat Hospital, Hamedan University of Medical Sciences, Hamedan, Iran

**Keywords:** Stressors, Mothers, Hospitalized children

## Abstract

**Objective:**

Hospitalization of a child can cause severe anxiety and stress in the parents, especially for the mother. This stress consequently affects the treatment course of the child. Hereby, we investigate the impact of different stressors in mothers of hospitalized children.

**Materials & Methods:**

In this cross-sectional study, 225 mothers of hospitalized children in the pediatric ward of Besat hospital were randomly selected and studied. Data collection tool was a two-part questionnaire gathered by interviewing the mother. The first part included demographic information of the patients.

The second part included questions regarding stressors in four different categories; child-related factors, environmental factors, socioeconomic factors and health professional factors. SPSS 16.5 was used for statistical analysis and data were analyzed by one way ANOVA and T test.

**Results:**

In the child-related factor category, fear of child death (84%); in the socioeconomic factor category, fear of disease in the other siblings (84%); in the environmental factor category, unpleasant odors in the ward (56%); and in the health professional category, not enough explanation about inserting IV lines, (54.2%) constituted the most important factors.

There was a meaningful correlation between the stressors and the mothers’ age and occupation, child age, days of hospitalization, types of admission and health insurance coverage, but there was no meaningful correlation between stressors and other factors.

**Conclusion:**

Professional and in depth training programs should be provided for health care providers and nursing staff regarding dealing with mothers of hospitalized children.

## Introduction

Illness and hospitalization are often critical events that a child is faced with (1) and the stress of it can affect all family members. Maternal stress and anxiety can also affect the child in two ways, transferring stress to the child and interfering with the mother’s ability of childcare. Currently, in many countries, given the importance of family-centered care, the mother stays at the child’s bedside for the entire time of hospitalization and participates in the process of taking care of the child (2-4).

The role of family-centered care in pediatric nursing, common understanding between the nursing staff and the child’s parents can lead to providing higher-quality of medical attention (5). Many aspects of the parents’ life will change during hospital stay, including their natural needs, and social and economic issues, which can cause stress and anxiety for the parents (6).

Feelings of stress and anxiety are often associated with the lack of information on diseases and medical procedures. The pain is caused by the imposed treatments, unfamiliarity with the hospital rules and regulations, unfriendly staff and being afraid of asking questions (7). Based on previous studies, factors that cause stress in mothers of hospitalized children are environmental factors, managerial factors; socioeconomic factors and factors that are related to the child’s circumstances (8). A higher level of family stress can reduce the ability of the mother to cope with problems (9). Nurses and parents have different perceptions of stressors in the child’s admission to a hospital. In other words, efforts that the hospital staff makes to reduce stress for parents may not be effective. It is not helpful and increases their stress levels too (10). Therefore, special attention should be given to identify the stressors in nursing care, planning and parents education, moving stressors and treatment in the same direction and the factors that can reduce the mother’s ability to provide childcare and delay in treatment progress (9). Despite the importance, in most hospitals there are no action plans or training programs to reduce stress for the parents and because of staff familiarity with the hospital environment, they do not assume that the hospital environment and setting can be a stress causing factor for the mothers of hospitalized children (11). Therefore, it is important to identify the stressors in hospitalized children and their impact on the treatment process and also to find out how these factors may be affected by different cultural background, ethnicity and the region. The decision was made to conduct this study at the pediatric ward in Besat hospital in the city of Hamedan.

## Materials & Methods

This study was a cross-sectional study performed on mothers of225 hospitalized children at the pediatric ward in Besat hospital, Hamedan in 2008. The sample size was determined considering a 95% confidence level and an 80% statistical power using simple random sampling without replacement on a limited population.

Data were collected using a two part questionnaire. The first part included the mother’s and the child’s demographic data (maternal age, maternal educational level, marital status, number of children, employment status, years of marriage, age and sex of the child, days of hospitalization, frequency of hospitalization, hospital admission criteria and types of insurance coverage), the second part of the questions was asking about the related stressors in four areas, children-related factors (11 questions), environmental factors (10 questions), socio-economic factors (7 questions) and factors related to hospital staff (11 questions). Researchers collected data through interviewing the mothers. Responses were categorized and rated according to a Likert scale of five degrees; no tension, low tension, moderate tension, high tension and very high tension on a scale of 1 to 5. Validities of the contents were used to determine the validity and a reliability coefficient of 0.97% was obtained for questions in the questionnaire. 

To determine the reliability, the questionnaire was completed through interviews with 20 mothers of hospitalized children at the pediatric ward in Besat hospital and then Cronbach’s alpha reliability was used.The reliability result obtained in different parts was more than 80 percent.

For collecting data, researchers visited pediatric wards; introduced themselves and explained their research goals for the mothers who wished to participate in the study, then began the interview by asking questions and completing the questionnaire. 

Sampling was conducted in the morning shift after completion of visits by the nursing staff if the mother was ready for the interview. A minimum of 24 hours of hospitalization was required for entering the study. After encoding, the collected data were analyzed using SPSS 16.5 for Windows (SPSS Inc., Chicago, Illinois) and then using descriptive statistics (frequency tables and percentages) and inferential statistics (ANOVA and t-test), the tables were constructed.

## Results

Most mothers (48.4%) were between 25 and 35 years of age and the least (9.3%) were above 35 years of age (Table 1). The education level of 80 (35.6%) mothers were elementary, 59 (26.2%) guidance education, 45 (20%) high school level, 28 (12.4%) were illiterate and 13 (5.8%) had university education. Two-hundred and twelve (94.2%) of the mothers had a husband and 13 (5.8%) had no husband. Two-hundred eighteen (96.9%) of the mothers were housewives and seven (3.1%) were employed. Regarding the years of marriage, 89 (39.6%) of the mothers had 5 to 10 years of marriage, 79 (35.1%) had less than 5 years and 57 (25.3%) had more than 10 years of marriage. Residential location; 153 (68%) of the mothers were living in villages and 72 (32%) were living in cities. Regarding the number of children, 96 (42.7%) of the mothers had only one child, 86 (38.2%) had two children and 43 (19.1%) had three or more children.

Age of admitted children in the order of the highest frequency to the lowest frequency; 95 children (42.8%) were under one years old, 88 children (39.1%) were 1 to 2 years old, 22 children (9.8%) were between 3 and 6 years old and 20 children (8.3%) were between 6 and 12 years old. One hundred and twenty-five (55.6%) of the children were female and 100 (44.4%) were male. One hundred and twelve cases (49.8%) were the first born child, 76 cases (33.8%) were the second born child and 37 cases (16.8%) were the third child. 

Seventy-eight cases (34.7%) had one day of hospitalization, 57 (23.1%) had two days, 29 cases (12.9%) had three days, 21 (9.3%) had four days and 45 cases (20%) were hospitalized for five days or more.

Regarding the number of hospitalizations, 148 (65.8%) were hospitalized for the first time, 45 patients (20%) for the second time and 32 (14.2%) were hospitalized for the third time or more. Concerning admission criteria, 125 (55.6%) were admitted as emergency inpatients and 100 (44.4%) of them were admitted as non-emergency inpatients. According to insurance coverage, 159 patients (70.7%) had insurance coverage and 66 (29.3%) had no coverage.

In the areas related to the children (Table 2), the most influential stressors for mothers of hospitalized children were fear of child death and the lowest one was concern about serum and other tubes that were connected to the child.

In areas related to social and economic factors (Table 3), the most influential stressors were fear from involvement of other children in their family and the lowest level of concern was about the distance and transportation problems to the hospital from their residential location or workplace.

In areas related to environmental factors (Table 4), the most influential stressors were the unpleasant odors in the ward and the lowest level of concern was about the equipments and instruments that were in the wards. 

In areas related to the staff (Table 5), the most influential stressors were inadequate explanation about medical procedures such as inserting IV lines and the lowest influential factors were giving the responsibility of monitoring IV serum to the mothers.

**Table 1:** Distribution of Mothers by Age

**Age**	**Frequency**	**Percent**
25-25	95	42.2
25-35	109	48.4
35 and more	21	9.3
Total	225	100

**Table 2:** Distribution of Stressors Related to Child Factors According to Likert Scale

**Questions**	**Very High**		**High**		**Average**		** Low**		**No Stress**		**Total**	
	**frequency**	**Percent**	**Frequency**	**Percent**	**Frequency**	**Percent**	**Frequency**	**Percent**	**Frequency**	**Percent**	**Frequency**	**Percent**
1- The child appears lethargic, weak and pale	186	82.7	39	17.3	0	0	0	0	0	0	225	100
2-Prolongation of hospitalization	134	59.6	62	27.6	17	7.6	12	5.3	0	0	225	100
3- The severity of disease	126	56	94	41.8	3	1.3	2	0.9	0	0	225	100
4- Child’s inability to eat	119	52.9	69	30.7	25	11.1	2	0.9	0	0	225	100
5- Fear of child death	189	84	29	12.9	7	Third	0	0	0	0	225	100
6- Uncertainty about future of the child’s medical condition	108	48	101	44.9	16	7.1	0	0	0	0	225	100
7- Fear of relapse	111	49.3	98	43.6	14	6.2	2	0.9	0	0	225	100
8- Child irritability and crying	116	51.6	55	24.4	31	13.8	15	6.7	8	3.6	225	100
9- Concern about serum IV fluid and different tubes connected to the child	82	36.4	46	20.4	21	9.3	39	17.3	37	16.4	225	100
10- Child’s pain	119	52.9	92	40.9	12	5.3	2	0.9	0	0	225	100
11- Concern about laboratory and imaging.	104	46.2	67	29.8	31	13.8	15	6.7	8	3.6	225	100

**Table 3:** Distribution of Stressors Related to Social and Economic Aspects According to Likert Scale

**Questions**	**Very High**		**High**		**Average**		**Low**		**No Stress**		**Total**	
	**Frequency**	**Percent**	**Frequency**	**Percent**	**Frequency**	**Percent**	**Frequency**	**Percent**	**Frequency**	**Percent**	**Frequency**	**Percent**
1- Failure to provide comfort to other children due to child illness	186	82.7	39	17.3	0	0	0	0	0	0	225	100
2- Problems related to drug availability	134	59.6	62	27.6	17	7.6	12	5.3	0	0	225	100
3- Inability to pay the costs of treatment and care	126	56	94	41.8	3	1.3	2	0.9	0	0	225	100
4- Concern about academic and school	119	52.9	69	30.7	25	11.1	2	0.9	10	4.4	225	100
5- Fear of other children having the same disease	189	84	29	12.9	7	Third	0	0	0	0	225	100
6- Fear of job loss because of the child’s disease	108	48	101	44.9	16	7.10	0	0	0	0	225	100
7- Being away from work and living place	99	44	48	21.3	29	12.90	31	13.8	18	8	225	100

**Table 4:** Distribution of Stressors Related to Environment According to Likert Scale

**Questions**	**Very High**		**High**		**Average**		**Low**		**No Stress**		**Total**	
	**Frequency**	**Percent**	**Frequency**	**Percent**	**Frequency**	**Percent**	**Frequency**	**Percent**	**Frequency**	**Percent**	**Frequency**	**Percent**
1- Noise pollution	99	44	48	21.3	29	12.9	31	13.8	18	8	225	100
2- Crowded room and the large number of children	94	41.8	59	26.2	45	20	8	3.6	19	8.4	225	100
3- Uncomfortable beds	72	32	63	28	51	22.7	28	12.4	11	4.9	225	100
4- Equipment	33	14.7	77	34.2	97	43.1	16	7.1	2	0.9	225	100
5- Concern about unpleasant odors	126	56	64	28.4	23	10.2	6	2.7	6	2.7	225	100
6- Unfamiliar environment	62	27.6	55	24.4	58	25.8	23	10.2	27	12	225	100
7- Lack of adequate sanitation and air pollution	122	54.2	71	31.6	20	8.9	4	1.8	8	3.6	225	100
8- No game room to entertain the children	49	21.8	45	20	81	36	22	9.8	28	12.8	225	100
9- No room to rest for mothers.	26	20.4	57	25.3	71	31.6	20	8.9	31	13.8	225	100
10- Shortage of blankets and bed liners	60	26.7	63	28	67	29.8	22	9.8	13	5.8	225	100

**Table 5:** Stressors Related to Hospital Staff According to Likert Scale

**Questions**	**Very High**		**High**		**Average**		**Low**		**No Stress**		**Total**	
	**Frequency**	**Percent**	**Frequency**	**Percent**	**Frequency**	**Percent**	**Frequency**	**Percent**	**Frequency**	**Percent**	**Frequency**	**Percent**
1- Inadequate explanation about the illness	121	53.8	77	34.2	10	4.4	13	5.8	4	1.8	225	100
2-Inadequate explanation about lab results and diagnostic procedures by physicians	107	47.6	95	42.2	15	6.7	6	2.7	2	0.9	225	100
3- Inadequate explanation by nursing staff about finding veins and other procedures.	122	54.2	91	40.4	10	4.4	2	0.9	0	0	225	100
4- Giving the responsibility for monitoring Serum IV fluids	37	16.4	18	8	22	9.8	52	23.1	96	42.7	225	100
5- Turning over responsibility for collecting samples to mothers by the nursing staff	38	16.9	6	2.7	12	5.3	21	9.3	148	65.3	225	100
6- Lack of attention to from nursing staff about mother’s problem	93	41.3	110	48.9	22	8.9	0	0	0	0	225	100
7 - Lack of proper nutrition for hospitalized children	86	38.2	118	52.4	8	3.6	6	2.7	7	Third	225	100

Statistically, there was a meaningful correlation between stressors and maternal age, mother’s occupation, child’s age, duration of hospitalization, types of admission and insurance coverage (0.001 ≥ p). There was no meaningful correlation between stressors and residential location, mother’s level of education, marital status and number of years being married, sex and birth order and how the child got admitted to the hospital.

## Discussion

In this study which was performed in the pediatrics ward of Besat hospital in Hamedan, the goal of the study was to determine the stressors in mothers of hospitalized children. The most influencing factor related to children’s care was fear of death and the least one was about serum IV fluid and other connected tubes to the child. In a study reported by Ismailzadeh, the most effective stressors related to child care was observation of the sick child by the mother and the least one was concern about the equipment (12). In the study performed by Miles and colleagues, the most effective stressors in areas related to the child were the child’s appearance and behavior and least one was the light condition and noise in the ward (13).

In this study, the most effective stressors related to socioeconomic factors were the fear of other children having the same problem and the least one was the distance from home and workplace. Lam et al. mentioned the cause of stress in the socioeconomic area was concern about the risk of disease for other children (14).

In a study conducted by Kristensson-Hallstrom, it was showed that invasive medical procedures are very stressful for some mothers and some mothers need emotional support during these procedures (4). 

In Ismailzadeh’s study, the highest socio-economic stressor was the mother’s worrying about other children left at home (12). There has been no comparable study about the least effective stressor in the socioeconomic area.

According to this study, the most effective environmental stressor was unpleasant odors and the least one was concern about equipment. In the study by Mwangi and colleagues, the most effective environmental stressors were crowded rooms, lack of food, poor sanitation and fear from transmission of infections from other children to their child (10). In the study carried out bySoderback and Christensson, mothers’ complaints related to environmental factors were about lack of sanitation services (9). There has been no comparable study about the least effective stressor related to environmental factors.

Most of the stressors related to the staff and employees were caused by an inadequate explanation of inserting IV lines and the least effective stressor was giving the responsibility of serum IV fluid monitoring to mothers. Mwangi et al. showed that mothers (in addition to their own basic needs such as nutrition and rest) expect, were involved in the decision-making process by the medical staff and also take adequate explanation about healing process and invasive procedures (10). Lam et al. also showed that most mothers were passionate in taking care of their child and giving partial responsibility of child care to mothers can improve their mood. This study also showed that most mothers need more communication and explanation from the nursing staff about different procedures and their role and contribution in medical procedures to provide better care for their children (14).

Soderback and Christenssonlso showed that most mothers (83%) wanted to obtain simplified explanation about the medical procedures and to be involved in painful procedures such as inserting IV lines and blood sampling (9).

Kristensson-Hallstrom stated that most mothers had recognized that illness and hospitalization of their children had an overwhelming psychological and emotional impact on their own behavior and they did not have enough control over their reactions. In this condition, mothers need understanding of their psychological and emotional problems and toleration by the nursing staff (4).

In this study, statistically, there was a meaningful correlation among maternal age and occupation, child’s age, days of hospitalization and insurance coverage in causing maternal stress.

There have been no comparable studies about these items.

**In conclusion, **the results of this research indicated that professional and in depth training programs should be provided for health care providers and nursing staff regarding dealing with mothers of hospitalized children.
